# Sleep loss diminishes hippocampal reactivation and replay

**DOI:** 10.21203/rs.3.rs-2540186/v1

**Published:** 2023-02-16

**Authors:** Bapun Giri, Utku Kaya, Kourosh Maboudi, Ted Abel, Kamran Diba

**Affiliations:** (1) Dept of Anesthesiology and Neuroscience Graduate Program, 1150 W Medical Center Dr, University of Michigan Medical School, Ann Arbor, MI 48109; (2) Dept of Psychology, University of Wisconsin-Milwaukee, PO Box 413, Milwaukee, WI 53201; (3) Department of Neuroscience and Pharmacology, Iowa Neuroscience Institute, University of Iowa, Iowa City, Iowa, USA,

## Abstract

Memories benefit from sleep, and sleep loss immediately following learning has a negative impact on subsequent memory storage. Several prominent hypotheses ascribe a central role to hippocampal sharp-wave ripples (SWRs), and the concurrent reactivation and replay of neuronal patterns from waking experience, in the offline memory consolidation process that occurs during sleep. However, little is known about how SWRs, reactivation, and replay are affected when animals are subjected to sleep deprivation. We performed long duration (~12 h), high-density silicon probe recordings from rat hippocampal CA1 neurons, in animals that were either sleeping or sleep deprived following exposure to a novel maze environment. We found that SWRs showed a sustained rate of activity during sleep deprivation, similar to or higher than in natural sleep, but with decreased amplitudes for the sharp-waves combined with higher frequencies for the ripples. Furthermore, while hippocampal pyramidal cells showed a log-normal distribution of firing rates during sleep, these distributions were negatively skewed with a higher mean firing rate in both pyramidal cells and interneurons during sleep deprivation. During SWRs, however, firing rates were remarkably similar between both groups. Despite the abundant quantity of SWRs and the robust firing activity during these events in both groups, we found that reactivation of neurons was either completely abolished or significantly diminished during sleep deprivation compared to sleep. Interestingly, reactivation partially rebounded upon recovery sleep, but failed to reach the levels characteristic of natural sleep. Similarly, the number of replays were significantly lower during sleep deprivation and recovery sleep compared to natural sleep. These results provide a network-level account for the negative impact of sleep loss on hippocampal function and demonstrate that sleep loss impacts memory storage by causing a dissociation between the amount of SWRs and the replays and reactivations that take place during these events.

Memories undergo continuous refinement following learning, in a process referred to as memory consolidation in which sleep plays a critical role. Sleep immediately after learning benefits memories^1^ and memories can be disrupted by even a few hours of sleep loss^2^. Studies have highlighted the particular importance of the hippocampus for sleep-dependent memory consolidation. However, the mechanisms through which memories are impacted by sleep loss have yet to be understood. At the cellular level, studies have identified molecular signaling events that are impacted by sleep loss, particularly in the first several hours. At the circuits level, oscillatory activities during sleep are hypothesized to strengthen, stabilize, and optimize memories. Hippocampal sharp-wave ripples (SWRs), which feature sharp-waves in the dendrites of CA1 pyramidal cells coupled with ripple oscillations (150–250 Hz) near the cell bodies, are widely considered to play a critical role in sleep-dependent memory processes. SWRs are observed more frequently in sleep after memory tasks^3^. Disrupting activity during these oscillations impairs memory^4,5^, while enhancing them improves memory^6^.

Why are hippocampal sharp-wave ripples so important to memory? A key characteristic of these signals is that they are generated in the CA3 region of the hippocampus and then produce intense spiking activity in the pyramidal cells and interneurons throughout the hippocampal formation^7,8^ and beyond^9,10^. Such synchronized activity drives synaptic plasticity in the connections between neurons associated with individual memories, thereby enhancing the signal to noise for storage and recall of those memories in the network^11,12^. In fact, both synaptic strengthening, via long-term potentiation^13,14^ and synaptic weakening, via depotentiation or long-term depression^15,16^, have been associated with SWRs. Moreover, the spiking activity during SWRs can be highly patterned to reactivate and replay activities initially expressed during learning and behavior in a temporally compressed manner akin to rapid rehearsal^17^. By generating such rapid rehearsals, SWRs can strengthen and stabilize spatial representations in the hippocampus^5,18^, as well as broadcast this signal to cortical and subcortical brain regions^8,9,19^ to transfer, transform, and consolidate memories^1^. While SWRs and their associated reactivations and replays are widely considered to play a key role in the memory consolidation process, remarkably nothing is known about how these events are impacted by sleep deprivation.

Here, we provide a detailed account of the impact of sleep loss on hippocampal oscillations and firing patterns, including sharp-wave ripples and associated reactivation and replay. We performed unit and local field recordings from large populations of hippocampal neurons over unprecedented (~ 12 h) durations, starting during sleep at the end of the dark cycle and extending through to exploration of a novel maze, and sleep or sleep deprivation followed by recovery sleep. We observed differences in the physiological characteristics of sharp-wave ripples during sleep deprivation as compared to natural sleep: the amplitude of sharp-wave and the power of the ripples were higher in natural sleep whereas the frequency of ripple oscillations was higher during sleep deprivation. However, the rate of sharp-wave ripples during sleep deprivation was similar or higher compared to natural sleep, indicating that the key hippocampal mechanisms for memory consolidation remain intact during sleep deprivation. Analysis of firing rates showed that both pyramidal cells and interneurons fired at higher rates during sleep deprivation, resulting in a negatively skewed log distribution in pyramidal cells compared to log-normal distributions typical of natural sleep. Analysis of firing patterns, however, revealed that reactivation and replay were negatively impacted by sleep loss. Whereas sleeping animals displayed robust reactivation in sleep following novel maze exploration, sleep-deprived animals displayed either no reactivation or reactivation that decayed at a faster rate. A similar impact was observed on multi-neuronal trajectory replays; fewer significant replays were observed during sleep deprivation compared to natural sleep. Remarkably, reactivation, but not replay, partially rebounded during the subsequent recovery sleep, potentially indicating homeostatic maintenance. However, the amount of reactivation in recovery sleep remained significantly attenuated compared to the levels seen during natural sleep.

Overall, our study reveals the impact of sleep loss on hippocampal sharp-wave ripple events and associated reactivation and replay, thereby elucidating the mechanism by which sleep loss can impair hippocampus-dependent memory consolidation.

## Results

**Figure 1:**
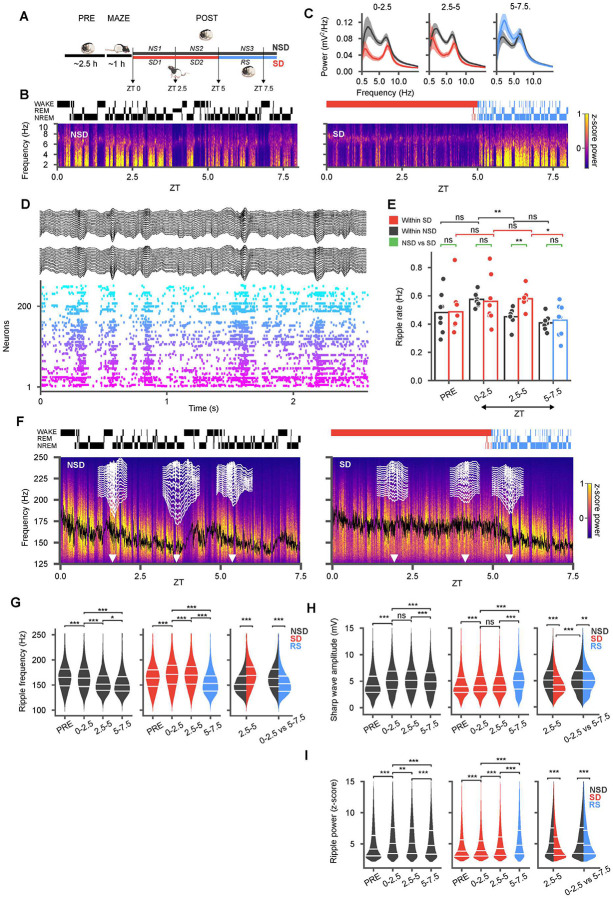
Sleep deprivation yields a similar amount of sharp-wave ripples but with lower amplitude sharp waves and higher frequency ripples compared to natural sleep. **(A)** After 2.5 h of rest and sleep in the home cage (PRE), animals were introduced to a novel track (MAZE) then returned to the home cage for either undisturbed sleep (NS1 and NS2), or 5h sleep deprivation (SD1 and SD2), followed by recovery sleep (RS). **(B)** Power spectral density (top right) in sample NSD (left) and SD (right) sessions from one rat with hypnogram (top) and spectrogram (bottom) of bandpass filtered (1–10 Hz) local field potential from CA1. **(C)** Average power spectral densities across all SD/RS (red/blue with corresponding shaded confidence intervals) and NSD (black with shaded confidence intervals) sessions in different blocks demonstrate suppressed spectral power during SD and a rebound in slow oscillations in RS. **(D)** Sample recording during sleep with local field potentials from two recording shanks (black, 16 channels each) along with rasters from simultaneously recorded units (arbitrary color and sorting). **(E)** Rate of ripples in various blocks compared between different NSD (black), SD (red) sessions, and RS (blue). Individual sessions are superimposed as dots over the bar plots. The rate of ripples decreases with sleep but remains elevated during sleep loss. **(F)** Power spectral densities in the ripple frequency band for the same sessions as in (B) with moving average of ripple frequency superimposed (black). Sample sharp-wave ripples (white traces across a 16-channel shank) at different time points (white arrow heads). **(G)** Violin plots across NSD (black) and SD/RS (red/blue) blocks show higher frequency of ripples in SD compared to NSD, with an undershoot in RS. Split violins in rightmost panel highlight cross-group comparisons for the second block of NSD vs SD and the first block of sleep (NS1 vs RS) in both groups. **(H, I)** Same as **(G)** for sharp-wave amplitude (H) and ripple band power (I) z-scored relative to session means (NSD/SD: each 8 sessions from 7 animals). Sharp-wave amplitudes and ripple power were lower in SD but partially rebounded in RS. (*p < 0.05; ** p < 0.01; *** p < 0.001)

We performed extracellular recordings from units and local field potentials using 128 channel high-density silicon probes (Diagnostic Biochips, MD) uni- and bilaterally implanted in the CA1 region of the rat hippocampus during behavior and sleep. Recordings initiated ~ 3.5 h before the onset of the light cycle with ~ 2.5 h of rest and sleep in a homecage (PRE). Animals were then placed in novel linear maze environments of differing shapes (MAZE) that they had not previously explored, and allowed to run for ~1h for water reward. Following the maze, animals were returned to the homecage for POST sessions that involved either natural sleep and rest (NSD) for ~9 h, or sleep deprivation (SD) via gentle handling for ~5 h followed by recovery sleep (RS) (Fig. 1A). We separated these periods into blocks of 2.5 h (e.g. NS1-NS3 vs SD1–2 & RS). SD and NSD sessions were carried out in pseudo-random order on different days spaced > 24 h apart, in the same animals (16 sessions in 7 rats). Units were identified based on automated and manual clustering and those that met strict criteria for stability were putatively classified into 754 pyramidal neurons (PN) and 96 interneurons (IN) using standard techniques (Methods). Power spectral calculations (Fig. 1B, C) demonstrated strong delta (<4 Hz) power in the hippocampal local field potential during natural slow-wave sleep and strong theta (5–10 Hz) during REM sleep. We did not see evidence for either prominent delta during sleep deprivation nor for prominent theta outside of REM periods^20^. However, we note that delta activity during sleep can spill over spectral power into neighboring theta frequency bins^21^. In our recordings, sleep deprivation was characterized by lower spectral power across frequencies. Recovery sleep following sleep deprivation subsequently featured a robust rebound in delta activity, consistent with models of sleep homeostasis^22,23^.

### A high rate of sharp-wave ripples is preserved during sleep deprivation.

Hippocampal sharp-wave ripple (SWR) complexes—sharp waves in the CA1 stratum radiatum accompanied by fast ripple oscillations (150–250 Hz) in the stratum pyramidale^24^—are observable during both awake and sleep states. Given the importance of SWRs for synaptic modifications of circuits in both the hippocampus and other brain regions^25^ and their hypothesized roles in sleep-dependent memory consolidation processes, we first focused on evaluating these events during our recordings (Fig. 1D). Previous studies have suggested that the incidence rate of ripples and associated population burst events play important homeostatic roles in hippocampal dynamics^15,16,26^. We therefore asked how the rate of these events change during sleep compared to a similar period during extended wakefulness. In naturally sleeping animals, we found that the incidence rate of SWRs decreased over time (Fig. 1E), consistent with a homeostatic effect from sleep (NS1 median = 0.57 Hz (interquartile range (IQR) = 0.06) vs NS2 median = 0.46 Hz (IQR = 0.03), *p* = 1.86 × 10^−3^, paired t-test (df=8)). In contrast, the rate of SWRs remained high in animals during sleep deprivation (SD1 median = 0.5 Hz (IQR = 0.16) vs SD2 median = 0.57 Hz (IQR = 0.02), *p* = 0.73, paired t-test (df = 8)) and was higher during the second block (zeitgeber time (ZT) = 2.5–5h) of SD compared to NSD (SD2 vs NS2, *p* = 1.07 × 10^−3^, t-test (df1 = 8, df2 = 8)). Once the SD animals were permitted recovery sleep (at ZT = 5 h), the rate of ripples dropped to levels lower than those in the early block of natural sleep (RS median = 0.45 Hz (IQR = 0.19) vs NS1 median = 0.57 Hz (IQR = 0.06), *p* = 7.87 × 10^−3^, t-test (df1 = 8, df2 = 8)). Overall, the number of sharp-wave ripples was not negatively affected by sleep loss but was rather higher during sleep-deprivation compared to natural sleep.

### Sleep loss alters the physiological properties of sharp-wave ripples

Given the prevalence of SWRs during both sleep and sleep deprivation, we hypothesized that other characteristics of these hippocampal events might differ across these periods. Differences in the physiological properties of SWRs have been observed in animal models of neurocognitive disorders^27–29^ and could reflect underlying circuit alterations. We therefore leveraged high-density electrodes in our recordings to measure and track changes in ripple frequency, ripple power, and the amplitudes of sharp waves across the duration of our recordings (Fig. 1F). Ripple oscillations in stratum pyramidale reflect rapid circuit dynamics that mediated by coupling between pyramidal cells and inhibitory interneurons^30,31,^see also^32^. The peak frequency of ripples in our recordings (Fig. 1G) decreased over the course of sleep, (NS1, median = 163.64 Hz (IQR = 34.85) vs NS3 median = 150.00 Hz (IQR = 28.79), *p* < 10^−10^, t-test (df1 = 41430, df2 = 29361)), but during sleep deprivation, ripple frequency remained elevated (Ripple frequency: NS1 median = 163.64 Hz (IQR = 34.85) vs SD1 median = 171.21 Hz (IQR = 37.88), *p* < 10^−10^, t-test (df1= 41430, df2 = 40381)) and was significantly higher compared to natural sleep, (NS2 median = 151.51 Hz (IQR = 28.78) vs SD2 median = 169.69 Hz (IQR = 34.84), *p* < 10^−10^, t-test (df1 = 32529, df2 = 41658)). The high frequency of ripples during the sleep deprivation period was also higher than those seen during PRE sleep. While changes in ripple frequency on the order of several Hz may be expected based on temperature differences across sleep and awake^33^, we observed larger differences of up to ~18 Hz (e.g. SD2 vs NS2: median = 169.69 Hz vs median = 151.51 Hz). Upon recovery sleep, ripple frequency dropped rapidly, to levels lower than during the similar sleep period in naturally sleeping animals (NS1 median = 163.64 Hz (IQR = 34.85) vs RS median = 153.03 Hz (IQR = 30.30), *p* < 10^−10^, t-test (df1 = 41430, df2 = 30767)), potentially reflecting the physiological impact of fatigue on the pyramidal cell-interneuron interactions that give rise to ripple oscillations.

The sharp waves concurrent with ripples reflect Schaffer collateral input from CA3 converging on the apical dendrites of CA1 neurons. The amplitude of these events therefore reflects the capacity of the CA3 network for synchronization. To better understand the impact of sleep and sleep loss we measured the amplitude of the sharp wave using the difference between the most negative deflection (typically in stratum radiatum) and the most positive deflection (typically in stratum oriens) recorded on our electrodes spanning CA1. In POST sleep we found increased amplitudes of sharp waves compared to PRE (NS1 vs PRE, median = 5.1 (IQR = 3.44) vs median = 4.13 (IQR = 3.03), *p* < 10^−10^, t-test (df1 = 41430, df2 = 30390)), which subsequently decreased over the course of natural sleep (NS1 median = 5.1 mV (IQR = 3.44) vs NS3 median = 4.87 mV (IQR = 3.35), *p* < 10^−10^, t-test (df1 = 41430, df2 = 29361)) (Fig. 1H). During sleep deprivation, on the other hand, the sharp-wave amplitudes were consistently lower than those in natural sleep (NS1 vs SD1, median = 5.1 mV (IQR = 3.44) vs median = 4.14 mV (IQR = 2.91), *p* < 10^−10^, t-test (df1 = 41430, df2 = 40378); NS2 vs SD2, median = 5.13 (IQR = 3.34) vs median = 4.18 (IQR = 2.88), *p* < 10^−10^, t-test (df1 = 32529, df2 = 41658)). In recovery sleep, sharp-wave amplitudes rebounded, but remained slightly lower than in natural sleep (NS1 median = 5.1 mV (IQR = 3.44) vs RS median = 5.05 (IQR = 3.279), *p* = 1.17 × 10^−3^, t-test (df1 = 41430, df2 = 30767)). The power of ripples (Fig. 1I) concurrent with the sharp-waves varied similarly to sharp-wave amplitude, indicating that higher amplitude sharp-waves in the stratum radiatum produce stronger ripples in the pyramidal layer. Ripple power (z-scored for relative to each session’s mean) was initially higher at onset of natural sleep (NS1 median = 5.07 (IQR = 4.12) vs PRE median = 4.16 (IQR = 3.26), *p* < 10^−10^, t-test (df1 = 41430, df2 = 30390)) and recovery sleep (RS median = 4.76 (IQR = 3.78) vs SD2 median = 4.22 (IQR = 2.92), *p* < 10^−10^, t-test (df1 = 30767, df2 = 41658)), but decreased over the course of sleep (NS1 median = 5.07 (IQR = 4.12) vs NS2 median = 5.10 (IQR = 4.09), *p* = 8.63 × 10^−3^, t-test (df1 = 41430, df2 = 32529)). These results demonstrate that while the total number of ripples remains elevated during sleep deprivation, sleep deprivation manifests with lower amplitude sharp waves and higher frequency ripples, potentially reflecting alterations in the interactions between excitatory and inhibitory cell populations during these events.

### Sleep loss disturbs firing-rate dynamics in the hippocampal network

**Figure 2:**
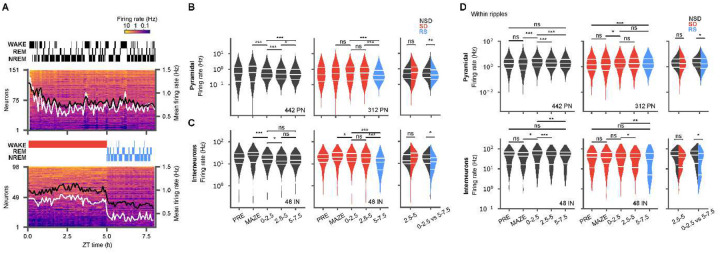
Hippocampal firing-rates are elevated and are more dispersed during sleep deprivation. (**A**) Two example sessions from non-sleep deprivation (NSD, top) and sleep deprivation (SD, bottom) with recovery sleep (RS), showing mean firing rates of pyramidal units (5 min bins, sorted by mean firing rate) and hypnograms during POST. Mean firing rates (right axis) for pyramidal cells are superimposed (white, this session; black, across all sessions). **(B)** Violin plots of firing rate distributions for pyramidal neurons during NSD (black; left, n = 7 sessions, 6 animals) and SD/RS (red/blue; middle, n = 8 sessions, 7 animals) in different blocks (PRE, MAZE, ZT 0–2.5, ZT 2.5–5, and ZT 5–7.5) show decreasing firing rates during sleep but elevated and more dispersed firing rates during SD. The total number of cells is noted in the lower right of each panel. Additional comparisons performed (right panel) between the second block of sleep deprivation (SD2) and the comparable period in NSD (NS2), as well as between the first block of sleep in each session, RS vs NS1, show an undershoot in firing during recovery sleep. **(C)** Same as (B) but for interneurons. **(D)** Same as (C) but for firing rates restricted to within ripples, demonstrating similar within-ripple firing rates in SD and NSD, but lower rates in RS (Wilcoxon signed rank tests for within group comparisons (left and middle panels), and Wilcoxon rank-sum tests for across group comparisons (right panels), * p < 0.05; ** p < 0.01; *** p < 0.001)

The firing rates of neurons are sensitive to changes in sleep states^34–36^ and serve as important signals of the homeostatic function of sleep^26,37,38^ and can reflect the strength of synaptic connectivity among neurons^16,38^. We therefore assessed the effects of sleep and sleep loss on hippocampal firing rate dynamics. During active exploration on the maze, the firing rates of pyramidal cells and interneurons increased significantly from PRE (PN *Δ*firing rate = 233 ± 35.71 %, *p* = 1 × 10^−4^; IN *Δ*firing rate = 127 ± 5.43 %, *p* = 1.36 × 10^−6^). However, following MAZE, sleep loss produced different dynamics from natural sleep (Fig. 2). Pyramidal cell firing rates (Fig. 2A, B) dropped significantly within hours of natural sleep (NS1 median = 0.51 Hz (IQR = 0.79) vs MAZE median = 0.62 Hz (IQR= 1.40), *p* = 7.72 × 10^−5^, Wilcoxon signed rank test (WSRT) (df1 = 442, df2 = 442)) and further over the course of the sleep cycle (NS1 median = 0.51 Hz (IQR = 0.79) vs NS2 median = 0.48 Hz (IQR= 0.75), *p* = 2.01 × 10^−7^, WSRT (df1 = 442, df2 = 442)), but in sleep deprivation, they remained elevated throughout the 5 h period (SD2 median = 0.57 Hz (IQR = 1.13) vs SD1 median = 0.57 Hz (IQR= 1.24), *p* = 0.18, WSRT (df1 = 312, df2 = 312)). Differences were also evident in the distributions of pyramidal cell firing rate, which were approximately log-normal during natural sleep^34,39^, but were heavily skewed away from the log-normal during sleep deprivation, with a broader distribution of firing rates compared with natural sleep (NS2 IQR = 0.62 log(Hz), *p* = 0.35, Shapiro-Wilk test (SWT) on log firing rates (df = 442) vs SD2 IQR = 0.82 log(Hz), *p* = 9.61 × 10^−3^, SWT (df = 312)); Fig. 2B). These negatively skewed distributions indicate that during sleep deprivation a few cells were active at substantially elevated firing rates, while other cells showed diminished firing, suggestive of competition among neurons^34^. In interneurons as well (Fig. 2C), firing rates decreased upon natural sleep and continued to decrease with further sleep (NS1 median = 16.13 Hz (IQR = 14.72) vs MAZE median = 24.43 Hz (IQR = 21.27), *p* = 3.97 × 10^−5^, WSRT (df = 48); NS2 median = 13.28 Hz (IQR = 15.44) vs NS1 median = 16.13 Hz (IQR = 014.72), *p* = 0.03, WSRT (df = 48)). Interneuron firing rates also decreased from MAZE to SD, but only slightly compared to NSD (MAZE median = 19.80 Hz (IQR = 18.46) vs SD1 median = 19.52 Hz (IQR = 16.75), *p* = 0.0143, WSRT (df = 48)), and remained stable for the remainder of SD. Overall, the increased firing rates and skewed distributions in sleep deprivation compared to natural sleep indicate a higher metabolic impact of prolonged waking, relative to sleep, on hippocampal activities, which confirm and extend previous observations^26,34^.

We next examined how these neuronal firing rates responded during recovery sleep. During recovery sleep, pyramidal cell firing rates decreased rapidly (RS median = 0.39 Hz (IQR = 0.65) vs SD2 median = 0.57 Hz (IQR = 1.12), *p* < 10^−10^, WSRT (df = 442)), in fact undershooting their levels compared to the first block of natural sleep sessions (NS1 median = 0.51 Hz (IQR = 0.79) vs RS median = 0.39 (IQR = 0.65) *p* = 3.20 × 10^−3^, Wilcoxon rank-sum test (WRT) (df1 = 442 vs df2 = 312)). A similar rapid firing rate drop was observed in interneurons (RS median = 11.87 Hz (IQR = 12.68) vs SD2 median = 18.76 (IQR = 19.51), *p* < 10^−10^, WSRT (df1 = 48, df2 = 48)), with significantly lower firing rates than during the first block of natural sleep (NS1 median = 16.13 Hz (IQR = 14.72) vs RS median = 11.87 Hz (IQR = 12.68), *p* = 0.0183, WRT (df1 = 48 vs df2 = 48)).

Interneurons of different types display a variety of firing response during SWRs and play an important role in determining the physiological characteristics of the ripple oscillation. Therefore, we also examined the firing responses of interneurons, alongside those of pyramidal cells, specifically within SWRs (Fig 2D). Interestingly, while firing rates within ripples varied across the periods we examined, we generally saw little difference between natural sleep and sleep deprivation (PN: NS2 median = 1.96 Hz (IQR = 3.16) vs SD2 median = 1.78 Hz (IQR = 3.83) *p* = 0.23, WRT (df1 = 442 vs df2 = 312); IN: NS2 median = 46.43 Hz (IQR = 54.79) vs SD2 median = 37.25 Hz (IQR = 45.72) *p* = 0.22, WRT (df1 = 48 vs df2 = 48)). However, we observed a significant decrease in the ripple firing rates of pyramidal cells and interneurons during recovery sleep compared to the similar period in natural sleep (PN: RS median = 1.68 Hz (IQR = 3.67) vs NS1 median = 2.09 Hz (IQR = 3.51), *p* = 0.04, WRT (df1 = 442 vs df2 = 312); IN: RS median = 29.34 Hz (IQR = 54.46) vs NS1 median = 49.70 Hz (IQR = 59.37) *p* = 0.01, WRT (df1 = 48 vs df2 = 48)). Notably, the interneuron firing rates during ripples appeared bimodal in recovery sleep, with a skew towards lower firing rates (median = 29.34 Hz (IQR = 54.46), *p* = 3.26 × 10^−4^, SWT on log firing rates (df = 48)). Some studies indicate that somatostatin positive interneurons generally fire at lower rates during SWRs than do other cells^40,41^. The firing rate skew we observe may therefore be accounted for by the differential impact of sleep loss specifically on this class of interneurons, consistent with a recent study employing immediate early genes^42^.

### Sleep loss attenuates memory reactivation

**Figure 3:**
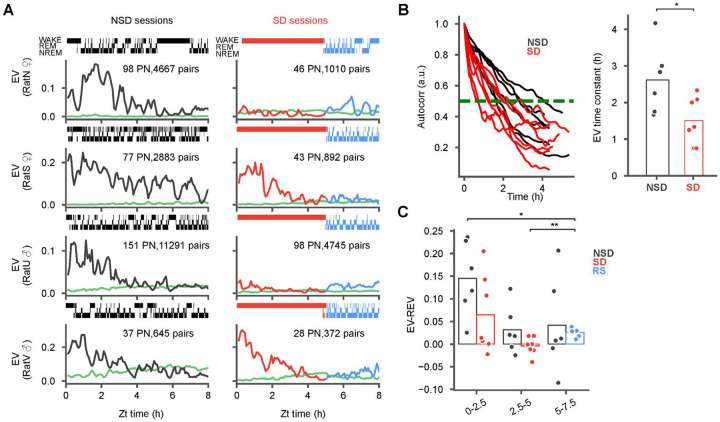
Reactivation attenuates during sleep deprivation and is not rescued by recovery sleep. **(A)** Explained variance (EV) of pairwise reactivation (NSD, black; SD, red) and its reverse (REV, green) during POST in natural sleep (NSD; left column) and sleep deprivation (SD) with recovery sleep (RS; right column) sessions from 4 animals (sex indicated on the y-axis). Shaded regions indicate low standard deviations. Additional sessions are provided in Extended Data Figure 1. NSD sessions feature robust reactivation lasting for hours while SD sessions show either some (rats S and V) or almost reactivation (rats N and U). **(B)** The EV auto-correlation (left panel) and corresponding time constants (right panel) derived from the half maxima (NSD: 5 animals, 6 sessions; SD: 6 animals, 7 sessions) demonstrate significantly faster decay in SD vs NSD. **(C)** Difference of EV and REV were calculated at ZT 0–2.5, ZT 2.5–5 and ZT 5–7.5, with markers for individual sessions superimposed. Note the significant increase between SD2 and RS, but significantly lower RS compared to NS1. (Wilcoxon signed rank tests for within group comparisons (panel C), and Wilcoxon rank-sum tests for across group comparisons (panel B) *p < 0.05)

Given that our results thus far demonstrate that SWRs and their overall population firing rates are largely preserved in SD, we next asked whether the specific content of SWRs may be impacted by sleep deprivation. We first examined the reactivation of neuronal ensembles, which have been linked to the memory function of the hippocampus^17,25^. Such reactivations can persist for hours after a novel experience^43^ and can broadcast the hippocampal signal to cortical regions^8,9,25^ To measure reactivation, we calculated the partial correlation explained variance (EV), which measures the similarity of pairwise correlations between MAZE and POST while controlling for pre-existing correlations in PRE^43–45^ in 250-ms bins in sliding 15-min windows (5 min steps; Fig. 3A). A time-reversed EV (REV) was used to estimate the chance level for reactivation^45,46^. In naturally sleeping animals following exposure to the novel maze we observed hours long reactivation, consistent with our previous study^43^. During sleep deprivation, however, we observed one of two scenarios: either virtually no reactivation (e.g. rats N and U, Fig. 3A; seen in 4 out of 7 sessions, Extended Data Figure 1) or alternately, reactivation somewhat similar to natural sleep but with a faster rate of decay (e.g. rats S and V, Fig. 3A; seen in 3 out of 7 sessions, Extended Data Figure 1). Pooled across subjects, the overall timescale of reactivation, estimated from the half-maximum of the EV autocorrelations (Fig. 3B), was significantly longer in sleep compared to sleep deprivation (NSD mean ± standard error of the mean (SEM) = 2.6 ± 0.38 h vs SD mean ± SEM = 1.5 ± 0.24 h, *p* = 0.0376, t-test (df1 = 6, df2 = 7). Remarkably, while reactivation was nearly absent at the end of the sleep deprivation period (Fig. 3C) it increased significantly at the onset of recovery sleep (Fig. 3C; RS mean (EV-REV) ± SEM = 0.026 ± .003 vs SD2 mean (EV-REV) ± SEM = 4.06 × 10^−3^ ± 7.56 × 10^−3^, p = 5.20 × 10^−3^, paired t-test (df = 7)). This suggests that the hippocampus is capable of reprising ensemble patterns reactivation even after a pause, such as during sleep deprivation. Nevertheless, the observed levels of reactivation during recovery sleep remained substantially lower compared to a similar period during natural sleep (Fig. 3C; RS mean (EV-REV) ± SEM = 0.026 ± .003, vs NS1 mean (EV-REV) ± SEM = 0.145 ± .033, *p* = 0.0156, t-test(df1 = 7, df2 = 6)), indicating a lasting outcome of sleep deprivation.

### Sequence replay deteriorates during sleep deprivation and recovery sleep

**Figure 4:**
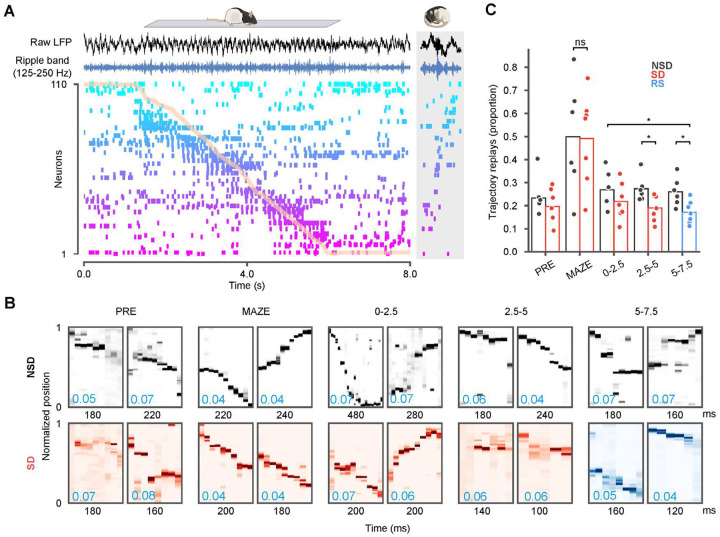
Trajectory replays deteriorate over sleep deprivation and recovery sleep. **(A)** Hippocampal spike raster and local field (LFP) during a sample run on the track (normalized track position overlaid in orange). Each row provides spike times for a single neuron, ordered by place field location. Raw LFP (black) and ripple-band filtered traces (blue) from one electrode are shown above the raster. The gray box on the right provides a sample replay sequence from POST sleep. **(B)** Two example trajectory replays shown for each of the PRE, MAZE, 0–2.5, 2.5–5, and 5–7.5 epochs. In each epoch, the sample events shown had traversed distances in the top 10 percentile and mean jump distance (blue text, lower left) across sequentially decoded bins in the lowest 10 percentile. **(C)** The proportion of candidate ripple events in different sleep (NSD) or sleep deprivation (SD) and recovery sleep (RS) epochs that decoded continuous trajectories. SD sessions featured significantly fewer trajectory replays by the second block. The proportion of replays in recovery sleep was significantly lower than the equivalent period in natural sleep (Wilcoxon rank-sum tests, *p < 0.05)

While pairwise measures, such as EV, measure neuronal reactivation, finer scale analysis has also revealed that neuronal activity during sharp-wave ripples can provide a temporally compressed replay of sequences of place cells that fired during maze behavior^47,48^. We observed similar replay sequences in our recordings as well (Fig. 4A). Most studies of sequence replay have been primarily directed at the brief periods of rest and sleep occurring within an hour of maze exposure. Taking advantage of our long duration recordings, we investigated how sequential replay unfolds over several hours of sleep in comparison with sleep deprivation. As quantification of these events can rely on different assumptions about the nature of replay^49,50^, we focused on using Bayesian methods (Fig. 4 A–B) to simply quantify the proportion of ripple events that decode continuous movement through the maze environment (i.e. “trajectory replays”). Ripple events featuring ≥ 5 active units, animal’s movement speed < 8 cm/s, and peak ripple power > 1 s.d. were considered candidates for further analyses (*see* Methods). We assessed trajectory structure using the distance between decoded locations in adjacent time steps, referred to as “jump distance”^51,52^. Ripple events with jump distance < 40 cm in at least three consecutive time bins were classified as trajectory replays, and we assessed the distribution of these events across epochs and conditions. The proportion of ripples that qualified as trajectory replays was highest on the maze in both experimental groups, consistent with previous reports^53,54^. However, the proportion of trajectory replays was significantly lower in SD sessions compared to NSD sessions in the last two blocks (NS2 mean ± SEM = 0.27 ± 0.023 vs SD2 mean ± SEM = 0.19 ± 0.021, *p* = 0.0213, t-test (df1 = 6, df2 = 7); NS3 mean ± SEM = 0.26 ± 0.023 vs RS mean ± SEM = 0.17 ± 0.018, *p* = 0.0178, t-test (df1 = 7, df2 = 6)). Importantly, even during recovery sleep, replays did not rebound to the comparative levels in natural sleep (Fig. 4C; RS mean ± SEM = 0.17 ± 0.018 vs NS1 mean ± SEM = 0.27 ± 0.033 *p* = 0334, t-test (df1 = 7, df2 = 6)). These results demonstrate that the loss of sleep immediately following novel experience negatively impacts the hippocampal replay of place cell patterns following novel maze exposure, which fail to rebound during recovery sleep.

## Discussion:

Here, we use long-duration recordings to define how sleep loss alters hippocampal firing patterns. Our observations of the effects of sleep deprivation on hippocampal oscillations and ensemble firing patterns have important implications for understanding the role of sleep and the negative impact of sleep loss on hippocampal function.

### Sleep deprivation induces smaller sharp-waves with higher frequency ripples

We observed distinct effects of sleep deprivation on the electrophysiological features of sharp-wave ripples. We found lower amplitude sharp waves coupled with lower power ripples during sleep deprivation compared to natural sleep. The amplitude of sharp-waves and power in the ripple frequency band are typically considered to reflect the synchrony and coherence of CA3 inputs converging on CA1 neurons. Higher amplitude/higher power events were reported to produce greater spiking in CA1 neurons^25,55^, and resonate more strongly throughout the hippocampal formation^8^. However, these studies did not separate effects according to the background sleep/awake state of the animal, whereas here the differences we report contrast the effects of enforced wakefulness with natural sleep. One recent study reported that awake sharp-wave ripples, despite featuring lower amplitude sharp-waves than during sleep, nevertheless have a larger impact on prefrontal cortical neurons^56^. Similar paradoxical effects were also recently reported for other brain regions, where lower amplitude sharp-waves produced larger neuronal responses in extra-hippocampal regions^8^. These observations therefore indicate that larger sharp-waves do not necessarily translate to greater activation in target regions. Additionally, at the level of the hippocampus, we note that firing rates during SWRs remained comparable between sleep deprived and sleeping animals despite differences in SWR features, suggesting that both low and high amplitude sharp waves generate approximately similar spiking responses in hippocampal neurons.

Alongside these sharp-wave differences, we observed parallel differences in the frequency of the ripple oscillations at the CA1 pyramidal layer. Higher frequency ripples were present throughout sleep deprivation and these ripples showed a progressive drop in frequency during natural sleep and recovery sleep. The ripple oscillation frequency likely reflects temporal interactions between pyramidal cells and interneurons, presumably basket cells that fire rapidly during SWRs^30,31^. Brain temperature can also affect ripple frequency by several Hz^33^, though not quite up to the 18 Hz differences observed in our recordings. These observations suggest that the frequency of ripple oscillations can serve as a useful proxy for sleep pressure measurable directly from the hippocampal LFP. In this context, higher frequency ripples potentially reflect the higher metabolism of the awake state^57^ which is progressively lowered and reset in sleep^26^. Differences in ripple frequency can also reflect differences in neuromodulatory tone, such as activation of GABA-A, 5-HT1A or muscarinic receptors^58–60^, or different routing of inputs to CA1, with higher frequency ripples reflecting the influence of CA2 during waking^61^, and lower frequency ripples reflecting input from the entorhinal cortex^25,62^. Interestingly, lower frequency ripples have also been associated with aging^63^ and have been recently reported in a rodent model of Dravet syndrome^64^ compared with healthy young controls, whereas ripple frequency increases after learning^65^, consistent with this postulated correlation of ripple frequency with higher metabolic cost.

### Extended wakefulness increases spiking and broadens firing rate distributions.

We also observed sustained high firing rates of both pyramidal cells and interneurons in the hippocampus during sleep deprivation, which stood in contrast to decreasing firing rates over the course of sleep, especially in recovery sleep. This extends upon our previous work by demonstrating that enforced wakefulness produces a similar effect to spontaneous waking on hippocampal firing rates^26^. These dynamics are also consistent with the reported effects of waking to increase and sleep to decrease firing rates in neocortical regions^35,37,38,66^. Moreover, we found that pyramidal cells displayed a wider negatively skewed distribution of firing rates during sleep loss compared to sleep. Such broadening of firing-rate distributions have been associated with higher activity of interneurons^34^, as we also see during sleep loss. These observations indicate that during enforced wakefulness interneurons actively regulate competition between pyramidal neurons and suppress the firing of some neurons at the expense of others^34,67^, whereas the balance shifts towards disinhibition during slow-wave sleep^34,68^. Recovery sleep following sleep deprivation was further characterized by significantly lower firing rates in both pyramidal cells and interneurons compared with regular sleep, indicating an enduring effect of enforced wakefulness consistent with fatigue. A recent study further reported that somatostatin positive neurons, a subset of which are lacunosum-moleculare projecting interneurons that gate entorhinal cortical input to CA1^69^ and fire at lower rates during SWRs^40^, are distinctly driven during loss of sleep^42^. Intriguingly, we saw negatively skewed firing rates of interneurons in ripples during sleep deprivation that remained skewed even in subsequent recovery sleep, which could reflect differential activation of these cell types.

The firing rate patterns we report appear consistent with “the synaptic homeostasis hypothesis”^70^ which conjectures that waking drives strengthened connectivity between neurons, while sleep drives synaptic downscaling. The progressive decrease in reactivation and replay over the course sleep may likewise be consistent with this hypothesis as the pathways providing reverberation of waking patterns are continuously reduced. On the other hand, the more rapid decline in replay and reactivation during sleep deprivation versus during sleep is not readily reconciled with a preferential role for waking in synaptic strengthening. If neurons that fire together indeed wire together during waking, they could be expected to show more robust reactivation (as reflected in co-activity) during this brain state if it is indeed most dedicated to synaptic strengthening. Another possibility, however, is that the strengthening during awake activity is promiscuous rather than specific to the firing patterns evidenced on the maze. In this scenario, waking during sleep deprivation may actively interfere with hippocampal reactivation by provoking the hippocampus to generate and learn new patterns inconsistent with the maze experience. Similarly, whereas it has been conjectured that sharp-wave ripples may serve to downscale synapses^16,26,71^, reactivation and replay were longer lasting during sleep compared to sleep deprivation, even though both states featured a similar incidence of SWRs. The background brain states against which SWRs occur, along with the hippocampal activation patterns that they produce, including the specific content of reactivation and replays, likely play an role in determining their effects on the hippocampal circuit and other brain regions^8,56,72^.

### Sleep loss impairs hippocampal reactivation and replay

Among the most significant findings uncovered in this study is that even though we observed a similar number of SWRs during sleep and sleep deprivation, the hippocampal reactivations and replays of the maze experience elicited during these events were diminished during sleep deprivation compared to sleep. In several influential models of sleep-dependent memory consolidation, hippocampal reactivations and replays work to consolidate memories by reprising patterns to strengthen the connections between the neurons associated to a memory^73–77^. In the most recent formulation of the synaptic homeostasis hypothesis, as well, reactivations and replays play a critical role by sparing indexed memories from synaptic downscaling to improve the signal to noise of important circuit connections^70^. Despite the consensus that these neuronal firing patterns play a critical role in the memory function of sleep, little has been known until now about how they are impacted by sleep loss. We measured reactivation using the EV measure, which reflects the similarity of pairwise co-firings of neurons to their co-firings during the novel maze exposure^44^, while controlling for co-activations that are present prior to maze exposure^43,45^, consistent with the Hebbian principle that assemblies formed during an experience continue to co-fire thereafter. Trajectory replays, on the other hand, relate the positions sequentially decoded using Bayesian inference to the sequence of locations that rats run through on the maze. Thus, replays presuppose the presence of reactivation, but reactivation could be present in the absence of replay, so long as active neurons fire in ensembles that are coherent with the maze experience^78,79^. In this study, we found that reactivation during natural sleep lasted for several hours, consistent with our recent report^43^. During sleep deprivation, on the other hand, we observed a bimodality, with some sessions showing virtually no reactivation, while others showed reactivation the decayed at a faster rate compared to during sleep. An intriguing possibility is that this bimodality reflects differences in resilience to the effects of sleep deprivation^80,81^. However, we did not see evidence for a similar bimodality in the amount of trajectory replays, which was significantly lower by the second half of sleep deprivation, compared to natural sleep. This difference could be due to the methodological differences in the measures used to capture reactivation and replay, making a direct comparison very difficult^50^. A potential contribution to such differences, however, could arise if pairwise co-activations during sleep-deprivation are reflective of the maze experience, without linked into multi-neuronal sequences that decode to trajectories spanning the maze environment^82,83^. Nevertheless, our study shows that both replay and reactivation, each associated with the memory function of sleep^77,84,85^, were negatively impacted by sleep deprivation.

### The rebound of reactivation during recovery sleep

Remarkably, we observed a partial rebound in reactivation during recovery sleep following sleep deprivation. This rebound suggests that despite the diminished reactivation during sleep deprivation, the hippocampus maintained a latent trace of the maze experience that was revived when the animals fell asleep. Importantly, however, this rebound was only partial, and reactivation during the > 2.5 h of recovery sleep did not reach the levels observed during natural sleep in non-deprived sessions. While it remains conceivable that rebound reactivation could continue to increase beyond the duration of our recordings, this appears unlikely, because the greatest synchrony consistent with reactivation is observed at the onset of sleep, rather than during later stages when rodent sleep tends to be more fragmented and reactivation patterns become more diffuse^26,43^. Notably, we also did not detect a similar rebound in trajectory replays. Overall, the absence of a complete rebound in recovery sleep is remarkable, because while most indices of brain health and function return to homeostatic levels following sufficient recovery sleep, memories, once impaired by sleep loss or otherwise do not typically recover^2,86–88^. It is noteworthy that cyclic AMP (cAMP) signaling that is prominent in the first several hours of sleep and is impaired by sleep deprivation is fully restored during recovery sleep^87,89^. Similarly full recovery is observed in the transcription of genes that are differentially impacted by sleep deprivation following recovery sleep^88^, in contrast to reactivation and replay as we report. An intriguing possibility is that the temporal overlap between molecular signaling and replays is the key prerequisite for the consolidation of memory. Sleep loss potentially dissociates these processes either by suppressing one or both processes during the deprivation period, or by allowing for a full rebound in cAMP or other molecular pathways but not reactivations and replays in the recovery sleep period.

Overall, our work calls attention to reactivation and replay as potentially crucial elements mediating the role of sleep in memory that are negatively impacted by sleep loss. The impairment of these neuronal firing patterns could destabilize hippocampal spatial representations^18^ and hippocampus-dependent spatial memories^5^. Furthermore, since SWRs provide privileged windows of communication between the hippocampus and the neocortex^90^, the impaired content of that communication is likely to have widespread impact on networks distributed throughout the brain^8,91^.

## Methods:

### Animals and surgical procedures

Four male and three female Long-Evans rats (300–500 grams) were used in this study. All surgeries were performed on isoflurane anesthetized animals head fixed on a stereotaxic frame. After removing hair from the head, the incision area was cleaned using alcohol and betadine. Next, an incision was made to expose the skull underneath. The skull was cleaned of tissues and blood, after which hydrogen peroxide was applied. Coordinates for probe implantation were marked above the dorsal hippocampus (AP: −3.36, ML: ±2.2) following measurement of bregma and lambda. Craniotomies were drilled at the marked location. Using a blunt needle, the dura was removed carefully to expose the brain surface. After cessation of bleeding, animals were implanted with 64 channel (8 shank “Buzsaki” probe; Neuronexus, MI; X animals) or 128 channel (8 shanks, Diagnostic Biochips, MD, 7-X animals) silicon probes. Ground and reference screws were placed over the cerebellum. Craniotomy was covered with DOWSIL silicone gel (3–4680, Dow Corning, Midland, MI) and wax. A copper mesh was built around the implant for protection and electrical shielding. All procedures involving animals were approved by the Animal Care and Use Committee at the University of Michigan.

### Behavior

Prior to the probe implant surgery animals were habituated to the experimenter for ≥ 40 mins for 5 days. Following habituation animals were water restricted and trained to associate water rewards with plastic wells. During the post-implant recovery period (7 days) animals were brought to the recording room for monitoring electrophysiology signals and probes were slowly lowered to the dorsal CA1 region of the hippocampus. In addition, animals were also habituated to sleep box for >1 h every day. Following this, animals were placed on a water restriction regiment for 24 h before experiments commenced. Each experimental session began by transferring animals to their sleep box ~4 h before the onset of light cycle. After 3 h of recording in the home cage, animals were transferred to a novel maze that they had not previously explored. These maze tracks were made distinct by the shape, color, and construction materials. Animals alternated for ~ 1 h between two water wells fixed at either ends of the maze to retrieve rewards from water wells. Following exploration, animals were transferred to the home cage and the recording continued for ≥ 10 h. Animals had access to *ad libitum* food and received ad libitum water for 30 mins per day.

### Sleep deprivation protocol

Sleep deprivation was performed at the onset of the light cycle in the home cage using a standard ‘gentle handling’ procedure^92,93^. Animals were extensively habituated to the experimenter conducting the sleep deprivation. During the initial hours of sleep deprivation, animals were kept awake by mild noises, tapping or gentle shaking of the cage when animals displayed signs of sleepiness. As sleep pressure built up over 5h sleep deprivation period, other techniques such as gently stroking the animal’s body with soft brush or disturbing bedding were increasingly employed to to ensure that animals stayed awake. Following sleep deprivation, animals were allowed to sleep and recover for 48 h before any further experiments.

### Data Acquisition

Electrophysiology data was acquired using OpenEphys^94^ or an Intan RHD recording controller sampled at 30 kHz. Analysis of local field potentials (LFP), was performed on signals downsampled to 1250 Hz. The animal’s position on the maze track was obtained using Optritrack (NaturalPoint, Inc, OR), which uses infrared cameras to locate a 3d markers that were clipped to the animal’s crown. Position data was sampled at either 60 Hz or 120 Hz and later interpolated for aligning with electrophysiology. Water rewards during alternation on the maze track were delivered via solenoids interfaced with custom built hardware using Arduino. The timestamps for water delivery were recorded via TTLs.

### Spike sorting and neuron type classification

All data went through filtering, thresholding and automatically sorting using SpyKING CIRCUS^95^, followed by manual inspection and reclustering using the Phy package (https://github.com/cortex-lab/phy/}. Only well isolated units were used in further analysis. Putative neurons were classified into pyramidal and interneurons based on peak waveform shape, firing rate, and interspike-interval. To ensure that a given neuron was reliably tracked across the recording duration, we divided each session into 5 equally sized bins (~2.5 h) and excluded any unit that fired below 25% of its overall mean in any given time bin. All LFP and unit analyses were performed using custom codes written in PYTHON and are available in our lab’s GitHub repository (https://github.com/diba-lab/NeuroPy).

### Sharp wave ripple detection and related properties

For detecting ripples, one channel from each shank were selected based on the (highest) mean power in the ripple frequency band (125–250 Hz). The Hilbert amplitude was averaged across all selected channels, then smoothed using a Gaussian kernel (σ = 12.5 *ms*) and z-scored. Putative ripple epochs were identified from timepoints exceeding 2.5 standard deviations (s.d.) and the start/stop was associated with signals > 0.5 s.d.. Candidate ripples < 50 ms or > 450 ms were excluded from further analyses. Sharp wave amplitudes were obtained from a bandpass (2–30 Hz) filtered LFP using the difference between maximum and minimum value across all recorded channels within a given ripple. The peak frequency of each ripple was estimated using a complex wavelet transform. The LFP was first high-pass filtered > 100 Hz. This filtered signal was then convolved with complex Morlet wavelets with central frequencies selected from linearly spaced frequencies in the ripple frequency band (100 to 250 Hz). Within each ripple, the frequency with maximum absolute wavelet power was designated as the peak ripple frequency.

### Sleep scoring

Sleep scoring was performed using correlation EMG, theta, and delta power. Correlation EMG was estimated by summing pairwise correlations across all channels calculated in 10 s time windows with a 1 s step^96,97^. For theta power, a recording channel with the highest mean power in the 5–10 Hz theta frequency band was identified. Following theta channel selection, the power spectral density was calculated for each window. Periods with low and high EMG power were labeled as sleep and wake, respectively. The theta (5–10 Hz) over delta (1–4 Hz) plus (10–14 Hz) band ratio of the power spectral density was used to detect transitions between high theta and low theta, using custom python software based on hidden Markov models followed by visual inspection. Sleep states with high theta were classified as rapid eye movement (REM) and the remainder were classified as non-REM (NREM). Wake periods with high theta were labeled as “active” and the remaining were labeled “quiet”. These labels were merged in WAKE for the main figures. All detected states went through additional visual inspection to correct any misclassifications.

### Explained variance measure for reactivation

Explained variance was calculated using previously described methods^43,44^ Briefly, spike times were binned into 250 ms time bins, creating an N byT matrix, where N is the number of neurons and T is the number of time bins. Pearson’s correlations, R, were determined for spike counts from neuronal pairs in 15 min sliding windows (window length 15 min, sliding 5 min steps) to produce P, an M-dimensional vector, where M is the number of cell pairs. To reduce spurious correlations arising from cross contamination of units from the same shank, only pairs with waveform similarity <0.8 were used. Next, to assess similarity between P vectors from different windows, the Pearson correlation R of these vectors (i.e., the correlation between cell pair correlations) was determined (e.g., R_[pre, post]_, R_[pre, maze]_ and R_[maze, post]_). Controlling for preexisting correlations in a given window (k) in PRE, the explained variance for a 15 min window (WIN) was calculated as:

$$\text{EV}(\text{WIN})={\left(\frac{R_{\left[\text{MAZE},\;\text{WIN}\right]}-R_{\left[\text{MAZE},\;\text{PRE}(k)\right]}\times R_{\left[\text{PRE}(k),\;\text{WIN}\right]}}{\sqrt{1-R_{\left[\text{MAZE},\;\text{PRE}(k)\right]}^{2}}\sqrt{1-R_{\left[\text{PRE}(k),\;\text{WIN}\right]}^{2}}}\right)}^{2}$$

averaged over all windows in PRE. To get an estimate of the chance level for EV, we calculated a time-reversed explained variance (REV) for each WIN^45,46^:

$$\text{REV}(\text{WIN})={\left(\frac{R_{\left[\text{MAZE},\;\text{PRE}\left(k\right)\right]}\times R_{\left[\text{PRE}(k),\;\text{WIN}\right]}}{\sqrt{1-R_{\left[\text{MAZE},\;\text{PRE}(k)\right]}^{2}}\sqrt{1-R_{\left[\text{PRE}(k),\;\text{WIN}\right]}^{2}}}\right)}^{2}$$

similarly averaged over PRE. To estimate the time constant of reactivation from each session^43^, we used the half-maximum of the autocorrelation function of EV.

### Place field calculations

Prior to calculating place fields, animals’ 2D positions were linearized using ISOMAP^98^ and visually inspected to ensure accuracy. For each unit, two firing rate maps were generated corresponding to each running direction. Occupancy within 2 cm spatial bins using timepoints when animal’s speed exceeded 8 cm/s were calculated and smoothed with a Gaussian kernel (sigma = 4 cm). For each neuron, spike counts within each spatial bin were determined and also smoothed with the Gaussian kernel (sigma = 4 cm). Then, each neuron’s firing rate map was generated by dividing the smoothed spike counts by the smoothed occupancy map. Neurons with peak firing rate < 0.5 Hz were excluded from further analysis.

### Decoding and sequence selection

Multiunit activity (MUA) was used to detect population burst events that are concurrent with sharp-wave ripples. Within a session, all putative spikes from all clusters were binned in 1 ms time bin and smoothed using a Gaussian kernel of *σ* = 20 ms. Candidate ripple events were identified if peak MUA activity exceeded 3 s.d.. The start and stop times were defined by extending the boundary to MUA above the mean. Two events occurring within 10 ms of each other were merged. Events with duration < 80 ms or > 500 ms were discarded.

Before decoding, candidate ripple events were required to satisfy 1) ≥ 5 active units, 2) movement speed < 8 cm/s, and 3) concurrent peak ripple power > 1 s.d.. For these analyses alone, to minimize decoding error, we included all stable clusters^99^. Position decoding was carried out on ripple events using Bayesian decoding^100^. Probabilities of the animal occupying each position bin *x*_*P*_ on the track were calculated according to:

$$P\left(x_{p}|n_{t}\right)=K_{t}\left\{\prod_{i=1}^{N}\lambda_{i}{\left[x_{p}\right]}^{n_{i,t}}\right\}e^{-\tau \sum_{i=1}^{N}\lambda_{i}\left[x_{p}\right]}$$

where *τ* is the duration of the time bin (20 ms) used, *λ*_*i*_[*x*_*p*_] is the firing rate of the *i*-th neuron at *x*_*p*_ *on* the maze, *K*_*t*_ is a normalization constant such that sum of probabilities across all position bins equals to 1 for each time bin, and *n*_*t*_ is the number of spikes fired by each neuron in that bin. Location with the maximum posterior probability in a given time bin was termed as that time bin’s ‘decoded location’. A candidate ripple event was classified as a ‘replay’ if it decoded a continuous trajectory across space for ≥ 60ms such that the distance between decoded locations in adjacent time bins was < 40cm. Posterior probability matrices for all ripple events that were classified as replay have been compiled in an interactive plot available in our github repository (https://github.com/diba-lab/sd_paper/trajectory_replay_events.html).

## Extended Data

**Extended Data Figure 1:**
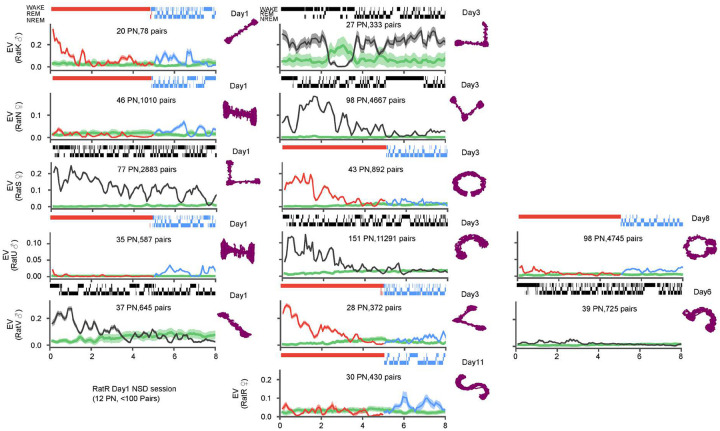
Temporal evolution of reactivation across all recorded sessions. Reactivation, measured using the explained variance (EV) metric, in thirteen sessions from six different animals (3 male and 3 female), as in Figure 3A. Each row provides session(s) from one animal, with number of putative pyramidal neurons and number of cellpairs used to calculate EV specified inside each panel. Hypnograms above panels depict sleep/wake history, with sleep deprivation/recovery sleep in red/blue and natural sleep in black Animals’ tracked position on the novel tracks (purple) are depicted on the right side of the panels with the day of the recording noted on top
